# Carmustine and methotrexate in combination after whole brain radiation therapy in breast cancer patients presenting with brain metastases: a retrospective study

**DOI:** 10.1186/1471-2407-10-257

**Published:** 2010-06-04

**Authors:** William Jacot, Marie-Cécile Gerlotto-Borne, Simon Thezenas, Stéphane Pouderoux, Sylvain Poujol, Mahdi About, Gilles Romieu

**Affiliations:** 1 Department of Medical Oncology, CRLC Val d'Aurelle, Montpellier, France; 2 Department of Biostatistics, CRLC Val d'Aurelle, Montpellier, France; 3 Oncopharmacology Department, CRLC Val d'Aurelle, Montpellier, France

## Abstract

**Background:**

Since 1999, patients presenting with brain metastases (BM) from breast cancer (BC) are treated in our institution with a carmustine (BCNU) - methotrexate (MTX) combination. We report here our clinical experience regarding this combination.

**Patients and Methods:**

Patients were treated by a combination of BCNU 100 mg/m² on day 1 and MTX 600 mg/m² on day 1 and 15 of a 28 day cycle. Treatment was continued until progression or unacceptable toxicity.

**Results:**

50 patients were treated between 1999 and 2007. 94% of the patients presented with concomitant extra-cerebral disease. Median number of previous metastatic setting chemotherapy regimens was 2 (0-5). Median number of cycles was 3 (1-20). There were 11 objective responses (23% [95%CI 12-37]) among 48 evaluable patients. Median progression-free survival and overall survival (OS) were 4.2 (95%CI: 2.8-5.3) and 6.9 (4.2-10.7) months respectively, with a one-year OS rate of 32% (20-46). Median Relative Dose Intensity for BCNU and MTX were 0.98 (0.31-1.1) and 0.96 (0.57-1.66) respectively. There were 2 presumed treatment-related deaths. One patient developed febrile neutropenia. Performance status, BS-BM score and presence of liver metastases were associated with OS in univariate analysis.

**Conclusions:**

This combination appears to be effective and well tolerated in good performance status BC patients presenting with BM.

## Background

Fifteen to 30% of patients with advanced breast cancer (BC) will develop brain metastases (BM) [1-6]. The vast majority of BC patients with BM have a concomitant extra-cerebral metastatic disease, pledging for systemic treatments. Central nervous system (CNS) metastases occur mainly within 2 years following a diagnosis of metastatic disease, with a 13 months median survival period from the diagnosis of BM in the HER-2 positive tumors setting [7]. Synchronous diagnosis of BC and BM is a rare event, with a 28-34 months median interval between primary diagnosis and the development of CNS involvement [5,8].

Prognosis of patients with multiple BM is poor, with a median survival of <1 year. There is currently no approved chemotherapy regimen for BM, and the current standard of care involves whole-brain radiation therapy (WBRT) and control of symptoms with steroids and anticonvulsants [3]. However, WBRT can be associated with a risk of neurotoxicity [9], and some patients refuse such treatment. Furthermore, even if some selected patients with recurrent metastases previously treated with WBRT can be candidates for salvage stereotactic radiosurgery, a majority of this population does not receive additional brain irradiation. In addition, the existence of an extra-cerebral disease precludes WBRT efficacy in term of overall cancer control, except in the uncommon cases of few (1-3) BM as the only metastatic lesions.

Various chemotherapy regimens have been investigated in BC patients with BM. However, standard agents used to treat BC, such as taxanes and anthracyclines, have demonstrated conflicting results regarding their efficacy in the treatment of BM [10,11]. The investigation of chemotherapy efficacy for the treatment of BM has been limited because of a presumed lack of effectiveness due to the blood-brain barrier (BBB). However, the importance of the BBB is probably overrated in the case of the neoangiogenesis surrounding macroscopic metastases or relapsed disease as the BBB has already been disrupted at this stage, resulting from the newly developed blood vessels not provided with the physiological properties of the common BBB. In such a setting, chemotherapeutic agents initially known not to cross the BBB, have been demonstrated to penetrate metastatic tissue [12] and so could be able to induce significant antitumor activity [11].

Methotrexate (MTX) is an active drug against breast and other primary cancers and can be effective when used at a high dose to reach the CNS and to achieve clinical activity [13]. Carmustine (BCNU) has demonstrated clinical activity in brain metastases from solid tumors [14]. Considering the lack of standard treatment validated in this population and the clinical activity of monotherapy methotrexate and BCNU in BM patients, these patients are treated in our Institution since 1999 with a combination of BCNU and MTX. We report here our clinical experience regarding the efficacy and safety profile of this combination.

## Patients and methods

### Patient Eligibility

Patients treated in our institution and affected by BC BM, diagnosed using either CT-scan or MRI of the brain, who received the combination of BCNU and MTX were considered in this retrospective study. Patients were identified from the Breast Cancer database of the Val d'Aurelle Medical Cancer Center, and records were reviewed for all patients treated with at least one cycle of BCNU-MTX for BC BM between 1999 and 2007. Identified patients were then followed until death or until February 2008. To be considered suitable for this treatment, patients had to have an Eastern Cooperative Oncology Group (ECOG) performance status (PS) [15] between 0 and 3. Patients were also required to have adequate bone marrow reserve (neutrophils ≥ 1.5.10^9^/L, platelets > 100.10^9^/L). This study was reviewed and approved by our Institutional Review Board.

### Treatment Plan

Patients were treated by the combination of BCNU and MTX according to the proposal of our in-site Breast Cancer Multidisciplinary Committee. Treatment was continued until progression, unacceptable toxicity, inter-current disease or patient's refusal to continue. The 4-week-based chemotherapy regimen consisted of a combination of BCNU 100 mg/m² and MTX 600 mg/m² on day 1 and MTX 600 mg/m² on day 15. A cycle restarted at day 28 pending hematological recovery (absolute neutrophils count ≥1.5.10^9^/L and platelets > 100.10^9^/L) and return to grade 0 or 1 for non hematological toxicity. If one or more of these conditions was not met, then a 1- to 2-week delay was allowed for recovery. A 50% dose reduction was applied to BCNU in case of severe toxicity (grade 4 neutropenia ≥ 7 days during the previous cycle; grade 3 or 4 febrile neutropenia; grade 3 or 4 infection with neutropenia; grade 4 anemia or thrombocytopenia or bleeding requiring transfusion, or any grade ≥ 3 non-hematological toxicity). The drug was discontinued in case of a second occurrence of severe toxicity. Methotrexate was administered on day 15 depending on hematological recovery and return to grade 0 or 1 for non-hematological toxicity. Urinary alkalization using sodium bicarbonate (in order to obtain a urinary pH > 7.5) was based on a 4 h 500 ml infusion of 4.2% sodium bicarbonate before MTX infusion, followed by a 12 h 1.5 l infusion of 1.4% sodium bicarbonate solution. Folinic acid supplementation (25 mg every 6 hours day 2 to 5 and day 16 to 19) was systematically used to minimize methotrexate hematological toxicity. Patients presenting with HER-2 over expressing tumors received in addition (when commercially available) trastuzumab injections at the dose of 4 mg/kg on days 1 and 15 of the same cycle. Granulocyte colony-stimulating factors were used as secondary neutropenia prophylaxis in case of delays in chemotherapy due to long lasting neutropenia. Concerning patients receiving endocrine treatment, the treatment was discontinued at the initiation of the chemotherapy combination.

### Study Assessments

Pretreatment evaluation included a complete medical history and clinical examination with tumor measurements (imaging studies and physical examination when appropriate), appropriate radiological tests, concomitant treatments, PS, and hematological and biochemical profiles. Tumor measurements were performed every other cycle during the treatment course and every 3 months subsequently until progression. During the treatment duration, complete blood counts including a platelet and leukocyte differential count were performed weekly.

### Response and Toxicity Criteria

CT-scans of the brain, chest, abdomen and pelvis were performed before the initiation of chemotherapy, then every 8 weeks (2 cycles) until disease progression or chemotherapy stop. For this retrospective analysis, patients were evaluated for response and progression according to the RECIST criteria [16]. A minimal duration of 4 weeks was required to document a response, and the best response was recorded for each patient. A patient with cerebral and extra-cerebral disease was considered as a responder if there was a significant (as per RECIST criterion) decrease of both cerebral and extra-cerebral lesions. Progressive disease was defined as cerebral and/or extra-cerebral progression of the disease. We considered clinical benefit as a disease stabilization lasting at least 6 months or an objective response. Progression-free survival was defined as the time from the date of BCNU-MTX treatment initiation until the date of progression or death. Survival was defined as the time from the date of treatment initiation to the date of death. Toxicity, graded according to the National Cancer Institute Common Toxicity Criteria (NCI-CTC, version 3.0), was assessed by clinical examination and biological tests before each cycle of chemotherapy.

### Statistical Considerations

Estimates of median progression-free survival, and overall survival with their 95% confidence intervals (CI) were calculated using the Kaplan-Meier product-limit method [17]. For toxicity analyses, the worst grade for each cycle of chemotherapy was used. Treatment compliance was evaluated for each drug by the Relative Dose Intensity (RDI), defined as the ratio of actual dose intensity for each drug (total administered dose per unit of time, expressed in mg/m²/week) to the planned dose per unit of time [18]. The relative dose intensity of BCNU and methotrexate by patient was calculated as the average RDI for all drugs. In addition, we performed an explanatory analysis to identify the prognostic factors affecting this population. Patients without clinical events at the date of February 29th, 2008 were censored at this date, excepted in cases of patients previously lost to follow-up. The statistical analyses were performed using STATA v9.0.

## Results

### Patients

From 1999 to 2007, 50 patients were treated with this combination. Patients' characteristics at the time of BM diagnosis are summarized in Table 1. Median age at the time of BM diagnosis was 54 years. Median time between diagnosis of BC and BM was 37 months (0-180). Only 1 patient presented with synchronous BM. Roughly two-thirds of the patient population was ECOG PS 0 or 1. No tumor was recorded to be Scarf, Bloom and Richardson (SBR) grade I, 40% were hormonal receptor negative and 50% of the 36 tumors analyzed for HER-2 amplification were positive. Ninety four percent of the patients presented with concomitant extra-cerebral disease, mainly bone (76%), liver (46%), lung (42%) and lymph node (26%) metastases. The median number of previous chemotherapy regimens in the metastatic setting was 2 (0-5). Twenty four patients (92% of the HR positive population) received previous hormonal therapy, either in the adjuvant or metastatic setting. The BM-associated clinico-radiological characteristics are summarized in Table 2. Six patients were clinically asymptomatic at the time of BM diagnosis. Only 5 patients presented with seizures and were treated with various anticonvulsants, precluding the analysis of an eventual interaction between concomitant medications and the chemotherapy efficacy and toxicity. No patients received prophylactic anticonvulsants. Thirty nine patients (78%) were treated by WBRT before the initiation of the BCNU-MTX chemotherapy (median dose 30 Gy, range 18 to 40). Due to the presence of cerebral and extra-cerebral disease, in order to treat extra-cerebral disease, the chemotherapeutic treatment was started shortly after the completion of the WBRT. Thirty three patients received planned chemotherapy immediately (less than one month after radiation therapy completion), and six patients received the combination chemotherapy as a salvage treatment after WBRT failure. Three patients (6%) had surgery and 2 patients were treated by radiosurgery (4%). Six patients were treated by the chemotherapeutic combination without previous radiotherapeutic or surgical treatment. Median time from BM diagnosis to initiation of chemotherapy was 1.3 months (range 0.1 to 8 months). Only 4 patients initiated chemotherapy more than 4 months (8 months) after the BM diagnosis.

**Table 1:** Patient characteristics at the time of BM diagnosis

Patient characteristics	Nb patients		%
Age at BM diagnosis (years)			
Median, range		54 (28-79)	
Time between initial diagnosis and BM diagnosis (months)			
Median, range		36.8 (0-180)	
Histology			
Lobular	6		12
Ductal	43		86
Other	1		2
SBR grade			
I	0		0
II	23		46.9
III	26		53.1
Not known	1		
Inflammatory breast cancer			
Yes	11		22.9
No	37		77.1
Not known	2		
Hormone receptor status			
ER-/PR-	19		40
ER-/PR+	1		4
ER+/PR-	18		40
ER+/PR+	7		16
Not known	5		
HER-2 status			
0	14		38.9
1^+^	2		5.6
2^+^	3		8.3
3^+^	17		47.2
Not known	14		
Number of previous chemotherapy lines			
Median, range		2 (0-5)	
ECOG Performance status			
0	19		38
1	13		26
2	14		28
3	4		8
Weight loss (%)			
Median, range		0 (0-15)	
RPA score			
1	2		4
2	44		88
3	4		8
BS-BM score			
0	1		2
1	21		42
2	26		52
3	2		4
Local relapse			
Yes	11		22.4
No	38		77.6
Extra cerebral metastases			
Yes	47		94
No	3		6
Extra cerebral metastases (sites)			
Bone	38		76
Liver	23		46
Lung	21		42
Lymph nodes	13		26
Others	9		18

**Abbreviations used**: BM: Brain metastases; RPA Score: Recursive partitioning analysis score; SBR Grade: Scarf Bloom and Richardson Grade; ER: Estrogen Receptor; PR: Progesterone Receptor; BS-BM: Basic Score for Brain Metastases.

**Table 2:** Brain metastases-associated characteristics

Brain Metastases characteristics	Number of patients		%
Number of brain metastases			
Median, range		4 (1-21)	
Brain metastases localization			
Supratentorial	13		26
Infratentorial	5		10
Supra and infratentorial	32		64
Associated symptoms			
Headaches, Nausea and Vomiting	38		76
Neurological deficiency	19		38
Seizures	5		10
Change in mental status	3		6

### Treatment Exposure

A total of 256 chemotherapy cycles were administered to 50 patients, with a median number of 3 (1-20) cycles and a 12.9 week median treatment duration (range 2-91). Thirty percent of the patients received up to 6 chemotherapy cycles, with 20% patients continuing the treatment after 6 cycles. Thirty four percent of the patients discontinued their treatment before the third cycle. The characterization of this population is explored on the prognostic factors section. Concerning the HER-2 over-expressed population, 8 patients received 48 cycles of the combination BCNU-MTX and trastuzumab.

Chemotherapy dose modifications and delays of BCNU and MTX are summarized in table 3. The median RDI for BCNU and MTX were 0.98 (0.31-1.1) and 0.96 (0.57-1.66) respectively. Two patients received a MTX dose of 1 g/m², thus explaining the upper MTX RDI limit of 1.66.

**Table 3:** Chemotherapy dose modifications and delays

	Cycles (n = 256)		Patients (n = 50)	
Methotrexate dose modification	35	13.7%	14	28%
BCNU dose modification	38	14.8%	14	28%
Any dose modification	55	21.5%	18	36%
Cycle delay	22	8.6%	10	20%

### Efficacy

**Figure 1:**
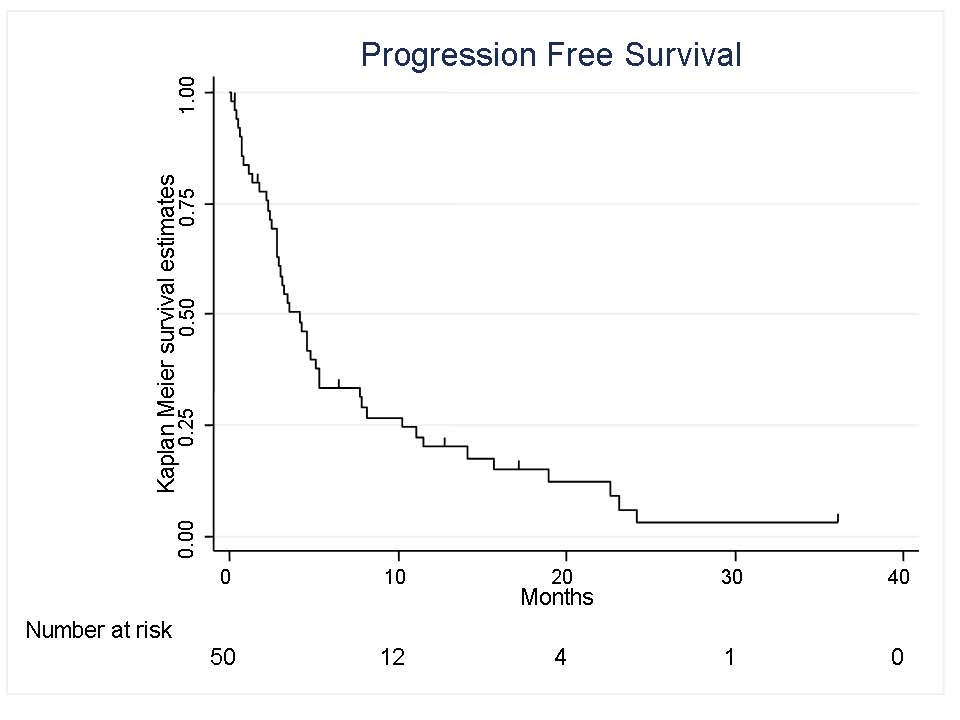
Progression Free Survival

**Figure 2:**
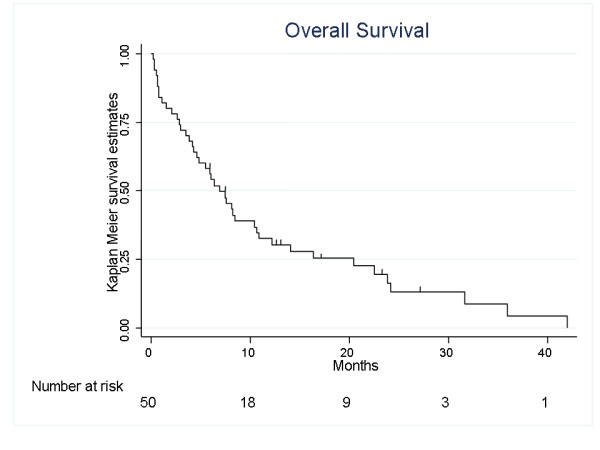
Overall Survival

There were 11 objective responses (23% [95%CI 12-37]) among 48 evaluable patients, with an additional 10% patients achieving disease stabilization lasting at least 6 months (5 patients), for a total clinical benefit rate of 33%. Two patients died of disease progression on the first 2 treatment weeks and were considered as non responders. Two patients were completely resected before the chemotherapy initiation and thus are not evaluable for response. Median response duration was 6.5 months (0.8 - 19.5). Median disease stabilization duration was 11.4 months (8.6 - 22.2+). Considering the HER-2 positive population treated by the BCNU-MTX trastuzumab combination (8 patients), 3 patients achieved a partial response and 2 additional patients achieved SD lasting more than 6 months. At the time of the current statistical analysis, 44 patients had a disease progression and 43 patients died. Thirty nine patients died from disease progression. In addition, there were 2 presumed treatment-related deaths (one case of acute renal failure at D6 of the first chemotherapy cycle, one patient developed a lethal febrile neutropenia 30 days after the fourth chemotherapeutic cycle). One patient was lost to follow-up, one patient died of an unrelated cause (myocardial infarction). Seven patients are still alive, 2 of them without evidence of residual disease. Median PFS was 4.2 months (95%CI: 2.8-5.3), with a one year PFS rate of 20.5% (10.3-33.2)(Fig. 1). First progression site was cerebral in 44% of the cases, outside the brain in 36%, and both intra- and extra-cerebral in 19% of the cases. Median overall survival of the whole population is 6.9 months (4.2-10.7), with a one-year OS rate of 32% (20-46) (Fig. 2). These survival data were 7.7 (0.4-NR) and 14.1 (0.4-NR) months respectively for PFS and OS in the population of 8 patients treated with trastuzumab and 3.5 (2.5-4.7) and 5.9 (3.9-8.2) months respectively for PFS and OS in the 42 patients population without trastuzumab treatment.

### Safety

Worst NCI-CTC toxicity grades experienced by patients during the chemotherapy cycles are given in Table 4. Myelosuppression was the most common toxicity. Neutropenia was observed in 56% of cycles; however grade 3-4 neutropenia was observed in only 16% of the cycles. One case of febrile neutropenia was recorded, leading one month later to the patient's death. However, the clinical presentation was unusual, with a long lasting neutropenia without complete recovery one month later. Grade 2-4 anemia was a rare event (16.5% of cycles). Severe (grade 3-4) thrombocytopenia was observed in only 4.8% of the cycles. No hemorrhagic episode was reported and no patient required platelets transfusion. Besides myelosuppression, adverse events (AEs) possibly or probably related to study drugs were usually mild in severity and manageable. The most common grade 2-4 extra-hematological AE was fatigue. Emesis was an uncommon event, with nearly 80% of the cycles with grade 0 emesis. Considering alopecia, the determination of this toxicity frequency and grade is difficult considering the high proportion of patients previously treated by whole brain radiation therapy. None of the patients treated by this chemotherapeutic combination without previous radiation therapy to the brain suffered from alopecia. There were no reports of chemotherapy-induced neurological side effects.

**Table 4:** Maximal toxicity (by chemotherapeutic cycle)

	Grade				
	
	0	1	2	3	4
**Anemia**	98 (39.4%)	110 (44.2%)	36 (14.5%)	4 (1.6%)	1 (0.4%)
**Neutropenia**	109 (43.8%)	53 (21.3%)	47 (18.9%)	31 (12.4%)	9 (3.6%)
**Leucopenia**	107 (43.0%)	44 (17.7%)	62 (24.9%)	33 (13.3%)	3 (1.2%)
**Thrombocytopenia**	155 (62.2%)	69 (27.7%)	13 (5.2%)	6 (2.4%)	6 (2.4%)
**Fever**	241 (99.6%)	0	1 (0.4%)	0	0
**Emesis**	187 (78.9%)	45 (19%)	5 (2.1%)	0	0
**Fatigue**	32 (13.4%)	169 (70.7%)	33 (13.8%)	5 (2.1%)	0
**Alopecia**	64 (33.0%)	22 (11.3%)	44 (22.7%)	64 (33%)	-

After treatment failure, 48% (24 patients) of the patients received additional chemotherapeutic or hormonal treatments (1 regimen for 11 patients, 2 regimens for 6 patients, 3 regimens for 3 patients, 4 regimens for 4 patients and 7 regimens for 1 patient) and one patient was lost to follow-up.

### Prognostic factors

Considering the relatively modest number of patients analyzed in this study, the exploratory analyses of prognostic factors affecting this population must be considered with caution.

**Figure 3:**
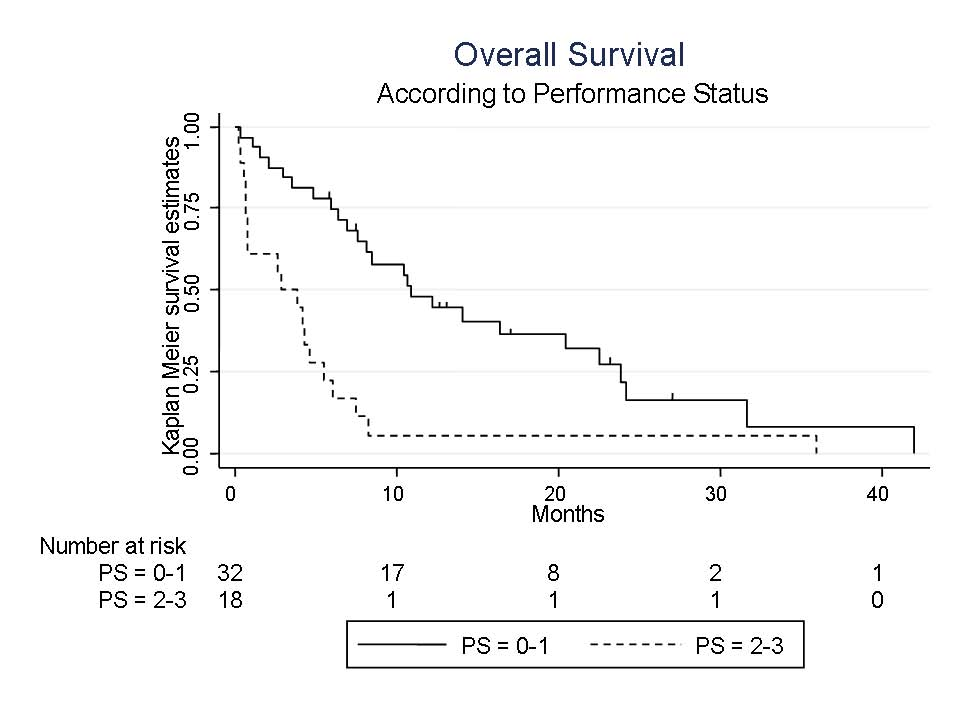
Overall Survival according to Performance Status

In univariate analysis, PS 0-1 versus 2-3, BS-BM (Basic Score for Brain Metastases) score 0-1 versus 2-3 and presence of liver metastases were the only factors significantly associated with prognosis. PS appears to be a strong prognostic factor, with a significantly higher 1-year survival rate (47.8% [30% - 60%] versus 5.5% [0% - 20%], *p *= 0.0002) and a longer median overall survival time (11.9 months [6.9 - 20.4] versus 2.9 months [0,7 - 4.7]) in the population of patients with PS 0 or 1 as compared to 2-3 (Fig. 3). Similarly, patients with a BS-BM score 2 or 3 had a significantly better prognosis than those with scores 0 or 1 (1-year survival 47% versus 14%, median overall survival 10.7 months [6.9 - 23.8] versus 3.9 months [0.75 - 5.49], respectively, *p *= 0.0007). Patients affected by liver metastases had a significantly worse prognosis than those without liver metastases (1-year survival 19.6% [6.3 - 38.2] versus 43% [24 - 60.7], median overall survival 3.9 months [1.1 - 8.2] versus 10.7 months [5.5 - 23.8], respectively, *p *= 0.0007). Neither hormone receptor status (negative versus one or both positive receptors) nor HER-2 status were found to be significantly associated with progression-free survival or overall survival (HER-2 status 0-1+ versus 2-3+: 1-year survival 28% versus 49%, median overall survival 6.4 months [2.9 - 31.6] versus 8.4 months [2.1 - 22.5], respectively, *p *= 0.77; 1-year progression-free survival 32% versus 15%, median progression-free survival 3.0 months [2.4 - 18.9] versus 5.1 months [2.2 - 8.1], respectively, *p *= 0.78). No statistical difference was observed for overall survival or progression-free survival between patients previously treated with 0-2 chemotherapeutic lines versus patients with 3+ previous chemotherapeutic lines. However, due to the small sample size of this study, significant and non significant results of the effects in particular subgroups needs to be interpretaed with extreme care. Considering this, a multivariate analysis was not performed.

At the same time, we tried to characterize the population of patients who were not able to complete the first 2 chemotherapy cycles due to toxicity, disease progression or performance status alteration. This population of 17 patients was affected with a really poor prognosis, with a median overall survival of one month [0.5 - 2.9]. Two-thirds of this population had a PS 2 or 3 and had BS-BM scores of 0 or 1. Also, liver metastases were present in 70% of the patients, a far more elevated percentage than in the whole study population.

## Discussion

BM of BC remains a clinical situation associated with poor prognosis, affecting mainly patients with metastatic disease outside the brain, and previously treated by several chemotherapeutic agents. In our study, patients previously received a median of 2 chemotherapy regimens before BM diagnossis. In addition, this clinical situation is a classical exclusion criterion of most clinical trials, precluding clinical data regarding the efficacy and toxicity of chemotherapeutic agents in this setting. However, the overall survival of our population is coherent with the ones reported by other clinical studies of third line chemotherapeutic agents in patients affected by metastatic BC without BM [13,19-22]. Prognosis after radiation therapy remain poor, with a median survival of 3 to 6 months and a 1-year survival of 15% [23,24]. This poor prognosis can be at least partially explained by the coexistence of an active extra-cerebral disease, remaining the main cause of death in this population [25]. These data, associated with the disruption of the BBB by the neoangiogenic switch associated with BM, supports the use of systemic treatment in patients affected with BM [11], in order to delay BM progression, to treat the concomitant extra-cerebral disease and to improve survival. A growing number of publications is supporting this idea, and the use of chemotherapy has even been identified as a prognostic factor in this population [24]. From a theoretical point of view, the BCNU-MTX combination, using non cross-resistant drugs not usually used in first or second line treatment of metastatic BC, was a good candidate for such a clinical setting.

Our population was affected with classical negative prognostic factors of metastatic BC, and of BM (young age, high SBR grade, high frequency of hormonal receptor-negative tumors, active extra-cerebral disease in 94% of the cases, altered PS) [11,26,27]. Only 2 patients can be classified in a good prognostic group considering RPA (Recursive partitioning analysis) or BS-BM classification. It is interesting to note that these 2 patients, as well as the 2 patients alive without evidence of intracranial disease, were in the radiation therapy group and that their BM were not surgically resected. However, considering such a poor prognostic group, in an unselected population, the results of this retrospective study are encouraging, with a clinical benefit rate of 33%, an overall response rate of 23% and a median overall survival of 6.9 months. These results compare favorably with other reported third line chemotherapeutic regimens in more selected patients not affected by BM [13,19-22]. In addition, it is important to note, while comparing to whole brain radiation therapy alone, that our survival data are calculated from the start of the chemotherapy cycles and that 89% of the patient population received previous radiation therapy, with a median time from BM diagnosis to initiation of chemotherapy of 1.3 months. Thus, the overall survival of our population from the time of diagnosis of BM must be considered longer, roughly equal to 9.1 months (5.2-13.2) in our study. All these data compares favorably with previous regimens reported in this setting of BM from BC (Table 5). Another important issue is the risk of over-evaluation of the clinical efficacy of a chemotherapeutic regimen initiated a few weeks after the end of the WBRT. However, 94% of the patients' population presented with extra-cerebral disease, and, to be classified as responder, a patient needed to have a significant response at the cerebral and extra-cerebral sites avoiding such a confounding effect of WBRT.

**Table 5:** Chemotherapeutic trials in breast cancer patients metastatic to the brain

Chemotherapy	N	Clinical study type	Objective response rate (%)	Progression-free survival (months)	Overall survival (months)	Reference
Cyclophosphamide, 5FU, methotrexate*	22	Prospective	59 (37-80)	NR	6.3 (0.5-20.8)	Boogerd et coll. (1992)[26]
Cisplatine etoposide	56	Prospective	38 (NR)	4,3 (0-71,8)	7.8 (0-71.8)	Franciosi et coll. (1999)[28]
Temozolomide capecitabine	24	Phase I	18 (NR)	3 (0.8-17.5)	NR	Rivera et coll. (2006)[29]
HD Methotrexate	32 (29 BC)	Retrospective	28 (NR)	NR	4.6 (0.7-31.2)	Lassman et coll. (2006)[13]
Capecitabine	7	Retrospective	28 (NR)	NR	NR	Ekenel et coll. (2007)[22]
Lapatinib**	39	Phase II	2,6 (0,2-26)	3 (2.3-3.7)	NR	Lin et coll. (2008)[34]
Lapatinib**	242	Phase II	6 (3.6-10.2)	2.4 (1.87-2.79)	6.37 (5.49-8.25)	Lin et col. (2009)[35]
Carmustin methotrexate (present study)	50	Retrospective	23 (12 - 37.3)	4.2 (2.8-5.3)	6.9 (4.2-10.7)	

* 2 patients received doxorubicin instead of methotrexate

** HER-2 overexpressed tumors

NR: Not reported

Considering the safety profile of this regimen, we were able to achieve a RDI greater than 90% for each of the 2 drugs. Dose reductions were applied in one third of the patients but there was no treatment discontinuation due to unacceptable toxicity. Clinically relevant toxicity is rare, and grade 3-4 hematological toxicity was rarely complicated. Only one patient presented a febrile neutropenia after 5 chemotherapy cycles, and died one month later due to a septic shock. The toxicity profile of the BCNU-MTX regimen appears rougly comparable with the aforementioned studies of chemotherapy in this setting [13,22,26,28,29]. In our opinion, this association can be considered as an option in BM BC patients previously pretreated by anthracyclins, taxanes and capecitabine.

However, in such a poor prognostic situation, it is important to identify factors associated with prognosis and with early treatment failure in order to avoid a useless and potentially toxic treatment and to target the population more likely to benefit from this regimen. One way to achieve this goal is to identify prognostic factors in this study. Use of prognostic index in patients suffering from brain metastases is an important issue in studies assessing the prognosis of this population. Our population of patients was analysed using two different stratification scores (RPA scoring system and BS-BM). RPA has been demonstrated as the most efficient scoring system in large populations [30]. However, when analysing our population of patients, there is a striking imbalance when using the RPA score (4%, 88% and 8% respectively of the patients were affected by RPA score I, II and III), precluding a reliable statistical analysis. Prognostic classification using the BS-BM scoring system allowed a more balanced distribution of the patients in two groups and was thus considered in our statistical analysis. PS was identified as a strong prognostic factor, together with BS-BM score. However, it is important to consider that PS is a part of the items used to calculate the BS-BM score, leading to a large redundancy between theses 2 univariate prognostic factors. A chemotherapeutic treatment does not seem indicated in BM BC patients with an altered PS, even more so if the patient is presenting with liver metastases. This poor prognosis population may be considered for best supportive care.

Another critical issue in this population is the influence of her-2 overexpression status on survival or disease free survival. In our study population, 55.5% of the patients with known HER-2 expression presented with HER-2 2+ or 3+ tumours, however Her-2 status had no statistical impact on survival or disease free survival. These results are conflicting considering other clinical studies in this setting. Eichler et al.[31] evaluated 83 BC patients with BM and concluded that HER-2 status was a strong predictor of survival in this population, with a reported prolonged survival of the HER62-positive population after the diagnosis of BM compared with HER-2-negative patients (17.1 months vs 5.2 months; P 5 .001). Nam et al.[32] have found in a population of 126 patients that metastatic BC who developed BM had higher proportions of triple-negative and HER2+/ER- tumour status. These data are in accordance with our results. In addition, they reported that HER-2 positive patients were affected with a more favourable clinical outcome than HER-2 negative patients, and more specifically that a significant survival benefit was noted in HER2-positive BM patients treated with trastuzumab when all 126 patients were compared in the following three groups: 21 HER2-positive patients who received trastuzumab after the onset of BM (12.8 months) vs 35 HER2-positive patients who did not receive trastuzumab after the onset of BM (4.0 months) vs 70 HER2-negative patients (3.4 months) (p = 0.0011). It is thus possible that the prognostic significance of the HER-2 status is linked to the therapeutic efficacy of trastuzumab-based treatments in this population. The differences between these 2 studies and our results can be related to the small sample size of our patient's population and to the limited use of trastuzumab in the HER-2-positive population (8 patients). These data, altogether with the expanded results of the Nam study reported by Park et al.[33] support the use of anti-HER-2 targeted therapies in BC patients with BM, even if they were previously treated with trastuzumab-based regimen.

## Conclusion

Despite the limitation of being a retrospective study, in this population of heavily pretreated patients affected by BC BM, the BCNU-MTX combination appears to be an effective and well tolerated combination. In the absence of standard chemotherapeutic treatment of BC BM, this combination appears to be a reasonable option in patients with good performance status.

## Competing interests

The authors declare that they have no competing interests.

## Authors' contributions

WJ, MA and ST performed the data analysis and quality assessment. MCGB was responsible for collecting the data and the data entry. WJ was the principal author. WJ, MCGB, StP and GR were the principal investigators. StP and SyP have made substantial contributions to the conception and design of the study and the interpretation of the data. WJ, MCGB, ST, StP, SyP, MA and GR have been involved in critically revising the manuscript and approval of the final version.

## Pre-publication history

The pre-publication history for this paper can be accessed here:

http://www.biomedcentral.com/1471-2407/10/257/prepub
